# Supplementary material for: Exploring dependence between categorical variables: Benefits and limitations of using variable selection within Bayesian clustering in relation to log-linear modelling with interaction terms
Source: J Stat Plan Inference. 2016 Jun;173:47–63. doi: 10.1016/j.jspi.2016.01.002 (PMC4896165; doi:10.1016/j.jspi.2016.01.002)
Supplement: MMC S1 — Additional information on: the calculation of the T matrices; the selection process of candidate edges for the model search algorithm; simulation parameters; the rate of accumulated mass of posterior model probability; inferences from an analysis based on pairwise odds-ratios. [file mmc1.pdf]

Supplemental Material

Exploring dependence between categorical variables: benefits and limitations of using variable selection within Bayesian clustering in relation to log-linear modelling with interaction terms

Michail Papathomas[1] and Sylvia Richardson[2]

[1] *School of Mathematics and Statistics, University of St Andrews, United Kingdom*

[2] *MRC Biostatistics Unit, Institute of Public Health, Cambridge, United Kingdom*

* Correspondence to: Michail Papathomas

The Observatory, University of St Andrews, Buchanan Gardens, St Andrews, Fife, Scotland, UK, KY16 9LZ, M.Papathomas@st-andrews.ac.uk

S1. A formal definition of interaction terms in a log-linear model

Denote with $\mathcal{P}$ the finite set of the P categorical covariates or factors. The resulting data can be arranged as counts in a P-way contingency table. A Poisson log-linear interaction model is a generalized linear model where the data are the cell counts of the contingency table. Denote with I_p the set of levels of factor $x_{\cdot p}$. Adopting the notation of Darroch et al. (1980), an individual cell is denoted by $\mathbf{l} = (l_p, p \in \mathcal{P})$, where l_p is an element of I_p. In other words, $\mathbf{l}$ is a vector of size P, with elements that describe the level of each factor. The cell count of $\mathbf{l}$ is denoted by $n(\mathbf{l})$. The cell counts are taken to be independent Poisson variables. The mean $\mu(\mathbf{l})$ of a certain cell is expanded so that,

$$\mu(\mathbf{l}) = \sum_{a \subseteq \mathcal{P}} \xi_a(\mathbf{l}_a).$$

Here, $\mathbf{l}_a = (l_p, p \in a)$ indicates a marginal cell defined by the levels of a subset a of $\mathcal{P}$. In the above sum, each term represents parameters ξ_a, one for each combination of levels of the factors in a. As pointed out in Dellaportas and Forster (1999), the ξ_a are equivalent to the parameters commonly used when a specific log-linear model is defined. When $a = \emptyset$, the ξ_a is

a constant. When $|a| = 1$, ξ_a is called a 'main effect'. When $|a| = m$, ξ_a is an interaction of order $m - 1$. To ensure identifiability, certain ξ_a parameters are apriori set equal to zero. If the number of individuals is fixed, say equal to n, the conditional distribution of the cell counts becomes multinomial and a constraint is added to the model to ensure that the total of the cell counts is n.

S2. Calculation of the $\boldsymbol{T}_\gamma$ matrices

In this and the next Section, we demonstrate in detail some technical aspects of our methodology. Throughout Sections S2 and S3 we use as a working example the real data analysis presented in Section 5.1 of the main manuscript, where six risk factors for coronary heart disease (CHD) were investigated.

To build the $\boldsymbol{T}_\gamma^{\text{Real data (CHD)}}$ matrix we follow the algorithm proposed in Section 3.3 of the main manuscript. Before reweighting so that the maximum element is one, we obtain,

$$\boldsymbol{T}_\gamma^{\text{Real data (CHD)}} = \begin{pmatrix} 0 & 721129 & \mathbf{725058} & \mathbf{129703} & \mathbf{504982} & 38648 \\ 0 & 0 & \mathbf{893535} & 143723 & \mathbf{670162} & 46371 \\ 0 & 0 & 0 & 145243 & 672967 & 46277 \\ 0 & 0 & 0 & 0 & \mathbf{111174} & 10221 \\ 0 & 0 & 0 & 0 & 0 & 41739 \end{pmatrix}$$

We reweight and obtain,

$$\boldsymbol{T}_\gamma^{\text{Real data (CHD)}} = \begin{pmatrix} 0 & 0.81 & \mathbf{0.81} & \mathbf{0.14} & \mathbf{0.56} & 0.04 \\ 0 & 0 & \mathbf{1} & 0.16 & \mathbf{0.75} & 0.05 \\ 0 & 0 & 0 & 0.16 & 0.75 & 0.05 \\ 0 & 0 & 0 & 0 & \mathbf{0.12} & 0.01 \\ 0 & 0 & 0 & 0 & 0 & 0.05 \end{pmatrix}$$

S3. A working example for the selection of candidate edges

Assume the currently accepted model in the reversible jump MCMC chain is 'ABC+DEF', and consider the $\boldsymbol{T}_{\gamma}^{\text{Real data (CHD)}}$ matrix.

Selecting an edge for possible addition

Elements $(1,4)$, $(1,5)$, $(1,6)$, $(2,4)$, $(2,5)$, $(2,6)$, $(3,4)$, $(3,5)$, $(3,6)$, of matrix $\boldsymbol{T}_{\gamma}^{\text{Real data (CHD)}}$ correspond to edges between the following pairs of factors: (AD), (AE), (AF), (BD), (BE), (BF), (CD), (CE), (CF). As stated at the start of this Section, we assume that the currently accepted model is 'ABC+DEF'. Therefore, these edges are not part of the currently accepted model, and one of them will become candidate edge for adding to the currently accepted model. The aforementioned elements of $\boldsymbol{T}_{\gamma}^{\text{Real data (CHD)}}$ can be arranged as,

$$\begin{pmatrix} - & - & - & \mathbf{0.14} & \mathbf{0.56} & 0.04 \\ - & - & - & 0.16 & \mathbf{0.75} & 0.05 \\ - & - & - & 0.16 & 0.75 & 0.05 \\ - & - & - & - & - & - \\ - & - & - & - & - & - \\ - & - & - & - & - & - \end{pmatrix}$$

We divide each element with the sum of all nine elements, obtaining,

$$\begin{pmatrix} - & - & - & \mathbf{0.0541} & \mathbf{0.2106} & 0.0161 \\ - & - & - & 0.0599 & \mathbf{0.2795} & 0.0193 \\ - & - & - & 0.0605 & 0.2795 & 0.0193 \\ - & - & - & - & - & - \\ - & - & - & - & - & - \\ - & - & - & - & - & - \end{pmatrix}$$

Now, the elements above add up to one. We sample an edge using them as probabilities associated with each one of the candidate edges. It is then proposed to add the selected edge to the currently accepted graphical model.

Selecting an edge for possible removal

Elements $(1,2)$, $(1,3)$, $(2,3)$, $(4,5)$, $(4,6)$, $(5,6)$, of matrix $\boldsymbol{T}_{\gamma}^{\text{Real data (CHD)}}$ correspond to

edges (AB), (AC), (BC), (DE), (DF), (EF). These edges form the currently accepted model, and one of them will become a candidate edge for removal. The aforementioned elements of $\boldsymbol{T}_{\gamma}^{\text{Real data (CHD)}}$ can be arranged as,

$$\begin{pmatrix} - & 0.81 & \mathbf{0.81} & - & - & - \\ - & & \mathbf{1} & - & - & - \\ - & - & - & - & - & - \\ - & - & - & - & \mathbf{0.12} & 0.01 \\ - & - & - & - & - & 0.05 \end{pmatrix}$$

To randomly select an edge for possible removal, we divide each element with the sum of all six elements, obtaining,

$$\begin{pmatrix} - & 0.2881 & \mathbf{0.2897} & - & - & - \\ - & - & \mathbf{0.3570} & - & - & - \\ - & - & - & - & - & - \\ - & - & - & - & \mathbf{0.0444} & 0.0041 \\ - & - & - & - & - & 0.0167 \end{pmatrix}$$

Now, the elements above add up to one. We sample an edge using these elements as probabilities associated with each one of the candidate edges. It is then proposed to remove the selected edge from the currently accepted graphical model.

Selecting two edges to swap

To attempt to swap one edge with another, we select a candidate edge for addition and one for simultaneous removal exactly as described above.

S4. Parameter coefficients and design matrices for simulated data

We present the parameter coefficients and design matrices for the five simulated data sets.

<u>Simulation 1</u>

$$X_1 = 0.097656 \times \begin{pmatrix} 1 & -1 & -1 & 1 & -1 & 1 & -1 & 1 & 1 & -1 & -1 & -1 & -1 & -1 & 1 & -1 & 1 & 1 & -1 \\ 1 & 1 & -1 & -1 & -1 & 1 & -1 & -1 & 1 & -1 & -1 & -1 & -1 & -1 & 1 & -1 & 1 & 1 & -1 \\ ... \\ 1 & 1 & 1 & 1 & 1 & 1 & 1 & 1 & 1 & 1 & 1 & 1 & 1 & 1 & 1 & 1 & 1 & 1 & 1 \end{pmatrix}$$

$$\theta_1 = (20, -1, 1, -1, 1, -1, 1, -1, 1, -1, 1, -1, 1, -1, 1, -1, 1, -1, 1)$$

.

$$X_2 = 0.097656 \times \begin{pmatrix} 1 & -1 & -1 & 1 & -1 & 1 & -1 & 1 & 1 & -1 & -1 & -1 & -1 & -1 & -1 & 1 & 1 \\ 1 & 1 & -1 & -1 & -1 & 1 & -1 & -1 & 1 & -1 & -1 & -1 & -1 & -1 & -1 & 1 & 1 \\ ... \\ 1 & 1 & 1 & 1 & 1 & 1 & 1 & 1 & 1 & 1 & 1 & 1 & 1 & 1 & 1 & 1 & 1 \end{pmatrix}$$

$$\theta_2 = (20, -1, 1, -1, 1, -1, 1, -1, 1, -1, 1, -1, 1, -1, 1, -1, 1)$$

.

$$X_3 = 0.097656 \times \begin{pmatrix} 1 & -1 & -1 & 1 & -1 & 1 & -1 & 1 & 1 & -1 & -1 & -1 & -1 & -1 & -1 & 1 & 1 \\ 1 & 1 & -1 & -1 & -1 & 1 & -1 & -1 & 1 & -1 & -1 & -1 & -1 & -1 & -1 & 1 & 1 \\ ... \\ 1 & 1 & 1 & 1 & 1 & 1 & 1 & 1 & 1 & 1 & 1 & 1 & 1 & 1 & 1 & 1 & 1 \end{pmatrix}$$

$$\theta_3 = (20, -1, 1, -1, 1, -1, 1, -1, 1, -1, 1, -1, 1, -1, 1, -1, 1)$$

.

Simulation 2

$$X_1 = 0.097656 \times \begin{pmatrix} 1 & -1 & -1 & 1 & -1 & 1 & -1 & 1 & 1 & -1 & -1 & 1 & -1 & 1 & 1 & -1 & -1 & -1 & -1 \\ 1 & 1 & -1 & -1 & -1 & 1 & -1 & -1 & 1 & -1 & -1 & -1 & -1 & -1 & 1 & 1 & -1 & -1 & -1 \\ ... \\ 1 & 1 & 1 & 1 & 1 & 1 & 1 & 1 & 1 & 1 & 1 & 1 & 1 & 1 & 1 & 1 & 1 & 1 & 1 \end{pmatrix}$$

$$\theta_1 = (20, -1, 1, -1, 1, -1, 1, -1, 1, -1, 1, -1, 1, -1, 1, -1, 1, -1, 1,)$$

.

$$X_2 = 0.097656 \times \begin{pmatrix} 1 & -1 & -1 & 1 & -1 & 1 & -1 & 1 & 1 & -1 & -1 & 1 & -1 & 1 & -1 & -1 & -1 \\ 1 & 1 & -1 & -1 & -1 & 1 & -1 & -1 & 1 & -1 & -1 & -1 & -1 & 1 & -1 & -1 & -1 \\ ... \\ 1 & 1 & 1 & 1 & 1 & 1 & 1 & 1 & 1 & 1 & 1 & 1 & 1 & 1 & 1 & 1 & 1 \end{pmatrix}$$

$$\theta_2 = (20, -1, 1, -1, 1, -1, 1, -1, 1, -1, 1, -1, 1, -1, 1, -1, 1)$$

.

$$X_3 = 0.097656 \times \begin{pmatrix} 1 & -1 & -1 & 1 & -1 & 1 & -1 & 1 & 1 & -1 & -1 & -1 & 1 & -1 & -1 & -1 \\ 1 & 1 & -1 & -1 & -1 & 1 & -1 & -1 & 1 & -1 & -1 & -1 & 1 & -1 & -1 & -1 \\ ... \\ 1 & 1 & 1 & 1 & 1 & 1 & 1 & 1 & 1 & 1 & 1 & 1 & 1 & 1 & 1 & 1 \end{pmatrix}$$

$$\theta_3 = (20, -1, 1, -1, 1, -1, 1, -1, 1, -1, 1, -1, 1, -1, 1, -1)$$

.

Simulation 3

$$X_1 = 0.097656 \times \begin{pmatrix} 1 & -1 & -1 & 1 & -1 & 1 & -1 & 1 & 1 & -1 & -1 & 1 & -1 & 1 & 1 & -1 & -1 & -1 & 1 & -1 & 1 & 1 & -1 \\ 1 & 1 & -1 & -1 & -1 & 1 & -1 & -1 & 1 & -1 & -1 & -1 & -1 & -1 & 1 & 1 & -1 & -1 & 1 & -1 & 1 & 1 & -1 \\ ... \\ 1 & 1 & 1 & 1 & 1 & 1 & 1 & 1 & 1 & 1 & 1 & 1 & 1 & 1 & 1 & 1 & 1 & 1 & 1 & 1 & 1 & 1 & 1 \end{pmatrix}$$

$$\theta_1 = (20, -1, 1, -1, 1, -1, 1, -1, 1, -1, 1, -1, 1, -1, 1, -1, 1, -1, 1, -1, 1, -1, 1)$$

.

$$X_2 = 0.097656 \times \begin{pmatrix} 1 & -1 & -1 & 1 & -1 & 1 & -1 & 1 & 1 & -1 & -1 & 1 & -1 & 1 & -1 & -1 & 1 & -1 & 1 & 1 & -1 \\ 1 & 1 & -1 & -1 & -1 & 1 & -1 & -1 & 1 & -1 & -1 & -1 & -1 & 1 & -1 & -1 & 1 & -1 & 1 & 1 & -1 \\ ... \\ 1 & 1 & 1 & 1 & 1 & 1 & 1 & 1 & 1 & 1 & 1 & 1 & 1 & 1 & 1 & 1 & 1 & 1 & 1 & 1 & 1 \end{pmatrix}$$

$$\theta_2 = (20, -1, 1, -1, 1, -1, 1, -1, 1, -1, 1, -1, 1, -1, 1, -1, 1, -1, 1, -1, 1)$$

.

$$X_3 = 0.097656 \times \begin{pmatrix} 1 & -1 & -1 & 1 & -1 & 1 & -1 & 1 & 1 & -1 & -1 & -1 & 1 & -1 & -1 & 1 & -1 & 1 & 1 & -1 \\ 1 & 1 & -1 & -1 & -1 & 1 & -1 & -1 & 1 & -1 & -1 & -1 & 1 & -1 & -1 & 1 & -1 & 1 & 1 & -1 \\ ... \\ 1 & 1 & 1 & 1 & 1 & 1 & 1 & 1 & 1 & 1 & 1 & 1 & 1 & 1 & 1 & 1 & 1 & 1 & 1 & 1 \end{pmatrix}$$

$$\theta_3 = (20, -1, 1, -1, 1, -1, 1, -1, 1, -1, 1, -1, 1, -1, 1, -1, 1, -1, 1, -1)$$

.

Simulation 4

$$X_1 = \begin{pmatrix} 0.137 & -0.274 & -0.137 & -0.274 & 0.548 & 0.274 & -0.137 & 0.274 & 0.137 & \ldots & 0.274 & 0.137 \\ 0.137 & 0.137 & -0.137 & -0.274 & -0.274 & 0.274 & -0.137 & -0.137 & 0.137 & \ldots & 0.274 & 0.137 \\ \ldots \\ 0.137 & 0.1371 & 0.274 & 0.137 & 0.137 & 0.274 & 0.274 & 0.274 & 0.548 & \ldots & 0.274 & 0.548 \end{pmatrix}$$

$$\theta_1 = (20, -1, 1)$$

.

$$X_2 = \begin{pmatrix} 0.137 & -0.274 & -0.137 & -0.274 & 0.548 & 0.274 & -0.137 & 0.274 & 0.137 & \ldots & 0.274 & 0.137 \\ 0.137 & 0.137 & -0.137 & -0.274 & -0.274 & 0.274 & -0.137 & -0.137 & 0.137 & \ldots & 0.274 & 0.137 \\ \ldots \\ 0.137 & 0.137 & 0.274 & 0.137 & 0.137 & 0.274 & 0.274 & 0.274 & 0.548 & \ldots & 0.274 & 0.548 \end{pmatrix}$$

$$\theta_2 = (20, -1, 1)$$

.

$$X_3 = \begin{pmatrix} 0.137 & -0.274 & -0.137 & -0.274 & 0.548 & 0.274 & -0.137 & 0.274 & 0.137 & \dots & 0.274 & 0.137 \\ 0.137 & 0.137 & -0.137 & -0.274 & -0.274 & 0.274 & -0.137 & -0.137 & 0.137 & \dots & 0.274 & 0.137 \\ \dots \\ 0.137 & 0.137 & 0.274 & 0.137 & 0.137 & 0.274 & 0.274 & 0.274 & 0.548 & \dots & 0.274 & 0.137 \end{pmatrix}$$

$$\theta_3 = (20, -1, 1)$$

.

Simulation 5

$$X_1 = \begin{pmatrix} 0.39 & -0.39 & -0.39 & 0.39 & -0.39 & 0.39 & -0.39 & 0.39 & -0.39 & -0.39 & -0.39 & -0.39 & 0.39 & 0.39 & 0.39 \\ 0.39 & 0.39 & -0.39 & -0.39 & -0.39 & -0.39 & -0.39 & -0.39 & -0.39 & -0.39 & -0.39 & -0.39 & 0.39 & 0.39 & 0.39 \\ \dots \\ 0.39 & 0.39 & 0.39 & 0.39 & 0.39 & 0.39 & 0.39 & 0.39 & 0.39 & 0.39 & 0.39 & 0.39 & 0.39 & 0.39 & 0.39 \end{pmatrix}$$

$$\theta_1 = (20, -1, 1, -1, 1, -1, 1, -1, 1, -1, 1, -1, 1, -1, 1)$$

.

$$X_2 = \begin{pmatrix} 0.39 & -0.39 & -0.39 & 0.39 & -0.39 & 0.39 & -0.39 & 0.39 & -0.39 & -0.39 & -0.39 & -0.39 & 0.39 & 0.39 \\ 0.39 & 0.39 & -0.39 & -0.39 & -0.39 & -0.39 & -0.39 & -0.39 & -0.39 & -0.39 & -0.39 & -0.39 & 0.39 & 0.39 \\ \dots \\ 0.39 & 0.39 & 0.39 & 0.39 & 0.39 & 0.39 & 0.39 & 0.39 & 0.39 & 0.39 & 0.39 & 0.39 & 0.39 & 0.39 \end{pmatrix}$$

$$\theta_2 = (20, -1, 1, -1, 1, -1, 1, -1, 1, -1, 1, -1, 1, -1)$$

.

$$X_3 \begin{pmatrix} 0.39 & -0.39 & -0.39 & 0.39 & -0.39 & 0.39 & -0.39 & -0.39 & -0.39 & -0.39 & -0.39 & 0.39 & 0.39 & 0.39 \\ 0.39 & 0.39 & -0.39 & -0.39 & -0.39 & -0.39 & -0.39 & -0.39 & -0.39 & -0.39 & -0.39 & 0.39 & 0.39 & 0.39 \\ \dots \\ 0.39 & 0.39 & 0.39 & 0.39 & 0.39 & 0.39 & 0.39 & 0.39 & 0.39 & 0.39 & 0.39 & 0.39 & 0.39 & 0.39 \end{pmatrix}$$

$$\theta_3 = (20, -1, 1, -1, 1, -1, 1, -1, 1, -1, 1, -1, 1, -1)$$

.

S5. Rate of accumulated mass of posterior model probability for Simulations 1,2 and 3

Figures S1,S2 and S3 show the rate of accumulated posterior model probability for Simulations 1,2 and 3 respectively, with respect to the top ten models. We run four replications for each analysis, averaging over the results. The analyses are based on 110000 iterations of the reversible jump MCMC sampler, thinned to 11000 iterations. The top ten models and associated posterior probabilities were calculated using the search algorithm that combines PDV and the cluster specific approach in a balanced manner. We see that this algorithm performs very well in Simulation 1, and also in Simulation 3. It is not performing as well for Simulation 2, although differences in Simulations 2 and 3 are not very pronounced. The PDV algorithm performs worse than the thee other algorithms that employ cluster specific information, over the first 2000 iterations or so. As a note of caution, we must stress that the limited number of replications (4) for each analysis, suggests that increased variability is associated with the information presented in Figures S1,S2 and S3.

S6. Additional analysis based on pairwise odds-ratios

To gain some intuition on the performance of the different approaches, we conducted an additional investigation using the two real data sets analyzed in the submitted manuscript. Instead of the proposed $\boldsymbol{T}_\gamma$ matrix, the evidence for the presence or absence of an edge is assessed using pairwise odds-ratios, and results are compared with results from the strategy we adopt in this manuscript. The odds-ratios were calculated by fitting a logistic regression between the two factors, where the preceding letter in alphabetical order was modelled as the outcome. The further the odds-ratio from 1, the stronger the pairwise relation between the two factors. If 1 is not contained in the 95% CI for the odds-ratio, we interpret this as a strong indication that the edge between the two factors is present. Note that quantifying the strength of the evidence for the existence of an edge provided by the pairwise strategy is not straightforward, as the calculated odds-ratio cannot be easily converted into a probability.

In the first real data illustration (Table S1, corresponding to matrix $\boldsymbol{T}_\gamma^{\text{Real data (CHD)}}$ in Section 5.1 in the main manuscript) the two methods (our strategy based on the $\boldsymbol{T}_\gamma$ matrix and the one based on odds-ratios) perform comparably. We note that the pairwise strategy does not clearly indicate that 'F' has only a main effect (since the odds-ratio for the 'BF' edge does not

include 1), thus creating a potential False Positive compared to our strategy.

The difference in the results from the two approaches is illustrated clearly in the second real data illustration. In Table S2 (corresponding to matrix $\boldsymbol{T}_\gamma^{\text{Real data (GE) (2nd run)}}$ in Section 5.2 in the main manuscript), we see that the pairwise strategy wrongly indicates that the 'DF' edge is absent, and thus would fail to provide evidence for a 'DEF' three-way interaction. (The p-value for the slope coefficient for the logistic regression between 'D' and 'F' is 0.23). Note that in all analyses we have performed using our strategy and the $\boldsymbol{T}_\gamma$ matrices, we never encountered the case where the $\boldsymbol{T}_\gamma$ matrix fails to capture the existence of an edge, either in a pairwise or a higher order interaction, failure which would be detrimental to a model search algorithm. We also note a prominent False Positive edge ('AB') indicated by the pairwise strategy.

This limited comparison was carried out to confirm empirically what could be anticipated by design, namely that an approach based on clustering will give different evidence for the existence of links compared to an approach based on pairwise testing, and that our approach has potential benefits for detecting higher order interactions.

References

Darroch, J.N., Lauritzen, S.L. & Speed, T.P. (1980). Markov fields and log-linear interaction models for contingency tables. *The Annals of Statistics* **8**, 522-539.

Dellaportas, P. & Forster, J.J. (1999). Markov chain Monte Carlo model determination for hierarchical and graphical log-linear models. *Biometrika* **86**, 615-633.

Kaufman, L., Rousseeuw, P.J. (2005). Finding Groups in Data: An Introduction to Cluster Analysis. Wiley Series in Probability and Mathematical Statistics. Hoboken, NJ, Wiley-Interscience.

Papathomas, M., Molitor, J., Hoggart, C., Hastie, D. & Richardson, S. (2012). Exploring data from genetic association studies using Bayesian variable selection and the Dirichlet process: application to searching for gene-gene patterns. *Genet. Epidemiol.* **36**, 663-674.

Table S1: First real data illustration. Pairwise odds-ratios and CIs. We display with bold font the values of elements that correspond to an existing edge in the most probable model shown in Section 5.1.

A	B	C	D	E	F
A	0.74	**1.63**	**0.73**	**1.48**	1.14
	(0.61,0.89)	**(1.35,1.96)**	**(0.60,0.87)**	**(1.23,1.78)**	(0.88,1.49)
B		**0.06**	1.06	**1.49**	1.33
		**(0.04,0.07)**	(0.88,1.28)	**(1.24,1.80)**	(1.02,1.74)
C			1.02	0.67	0.94
			(0.85,1.23)	(0.56,0.81)	(0.72,1.23)
D				**1.14**	1.15
				**(1.16,1.69)**	(0.88,1.50)
E					1.26
					(0.96,1.64)

Table S2: Second real data illustration. Pairwise odds-ratios and CIs. We display with bold font the values of elements that correspond to an existing edge in the most probable model shown in Section 5.1.

A	B	C	D	E	F
A	7.50	0.93	1.03	0.96	1.01
	(6.20,9.12)	(0.78,1.11)	(0.85,1.24)	(0.82,1.14)	(0.86,1.20)
B		1.00	1.03	1.00	1.03
		(0.87,1.14)	(0.89,1.19)	(0.88,1.13)	(0.91,1.17)
C			0.94	0.94	1.12
			(0.81,1.09)	(0.82,1.07)	(0.99,1.28)
D				**0.40**	**0.91**
				**(0.35,0.46)**	**(0.79,1.05)**
E					**0.40**
					**(0.36,0.46)**

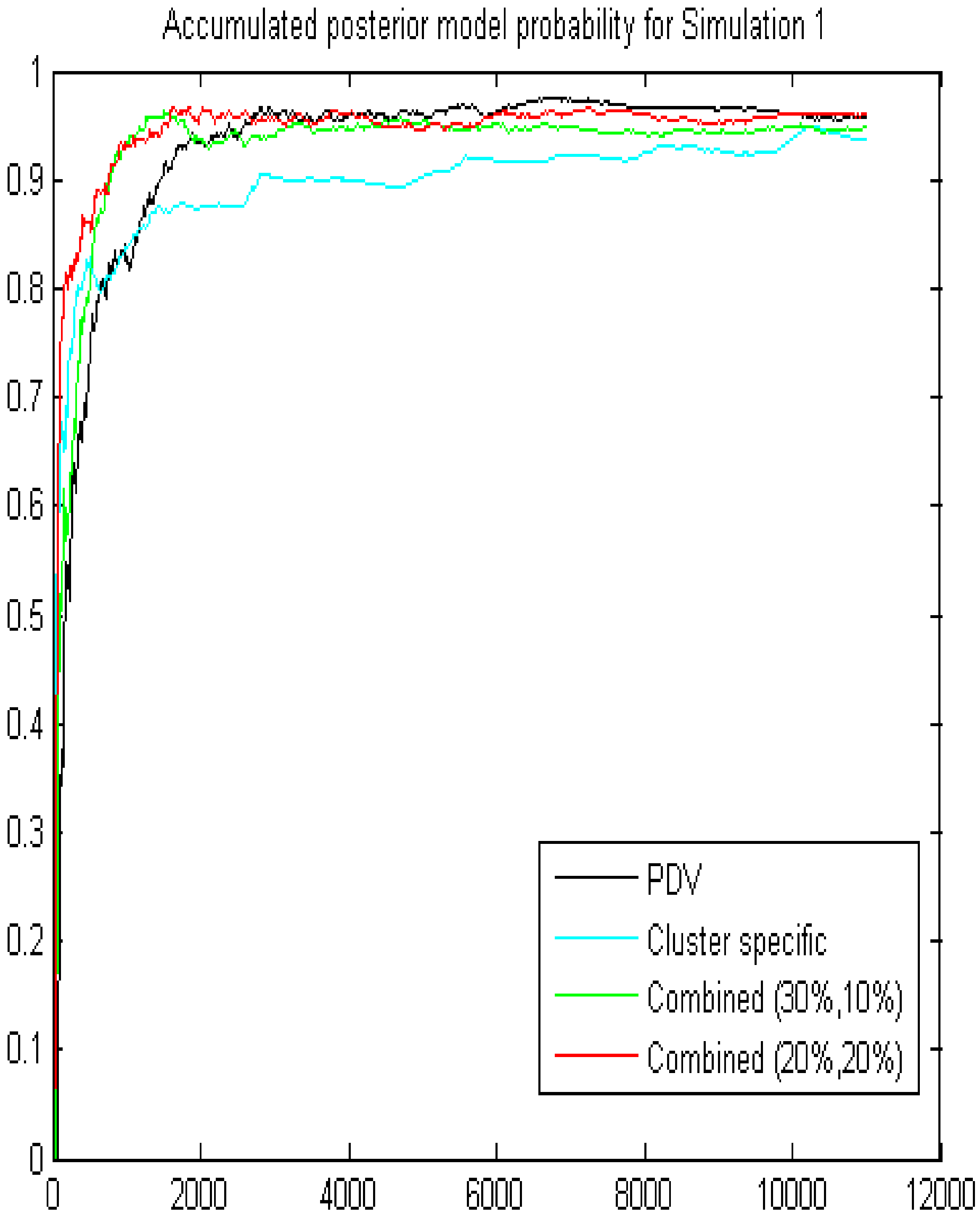

Figure S1: Rate of accumulated posterior model probability for Simulation 1, based on 110000 iterations, thinned to 11000 iterations.

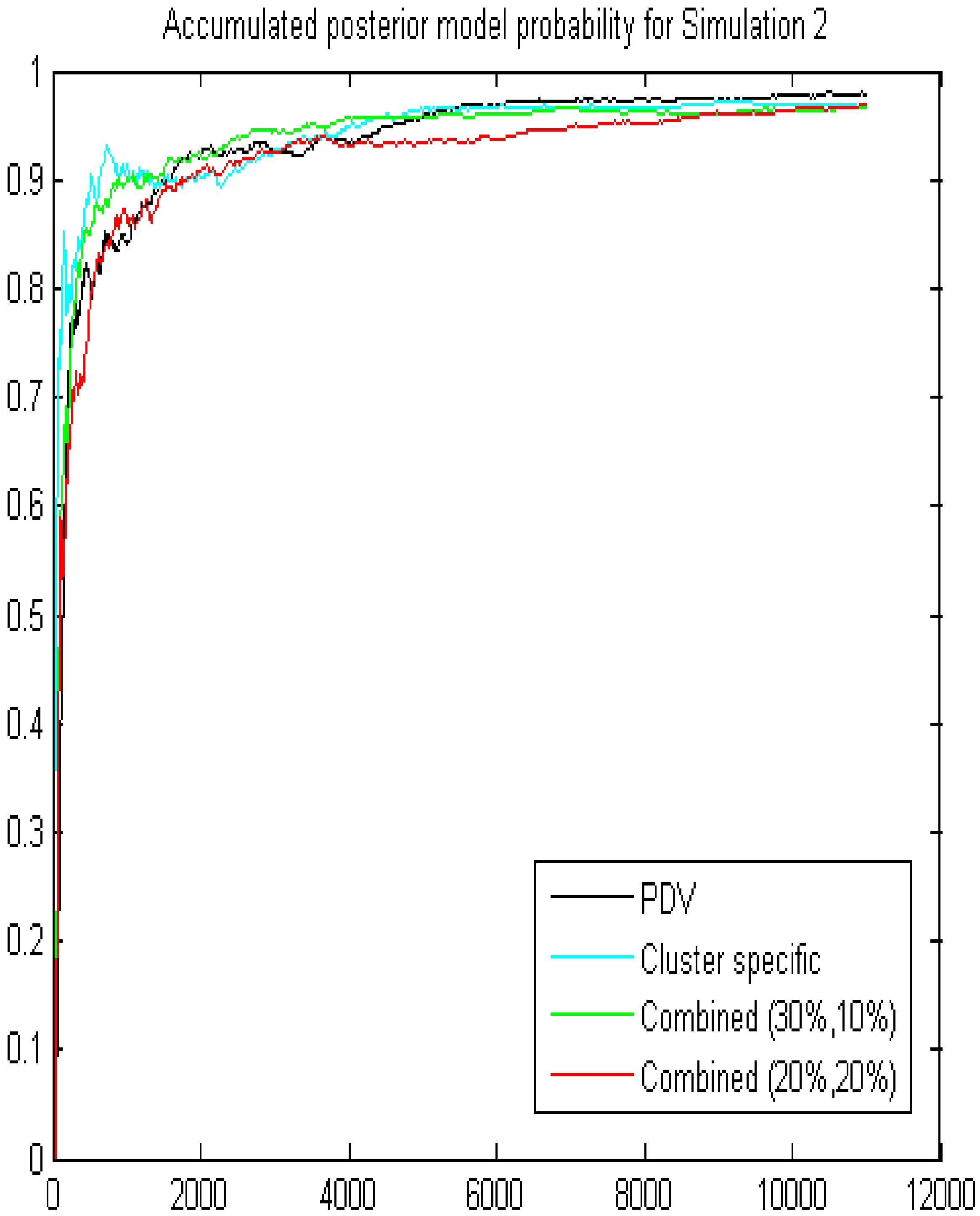

Figure S2: Rate of accumulated posterior model probability for Simulation 2, based on 110000 iterations, thinned to 11000 iterations.

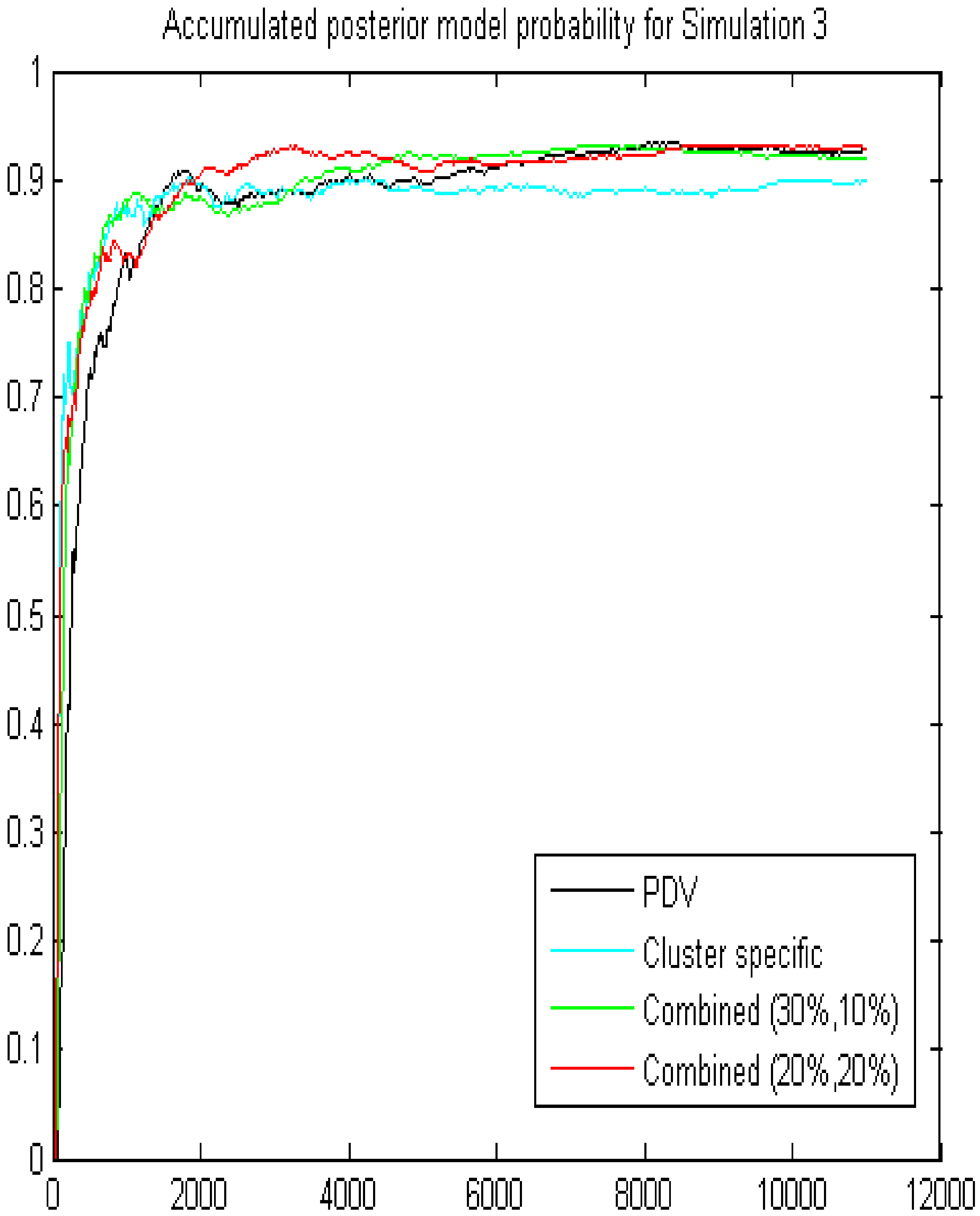

Figure S3: Rate of accumulated posterior model probability for Simulation 3, based on 110000 iterations, thinned to 11000 iterations.
